# Mitochondrial-Targeted Decyl-Triphenylphosphonium Enhances 2-Deoxy-D-Glucose Mediated Oxidative Stress and Clonogenic Killing of Multiple Myeloma Cells

**DOI:** 10.1371/journal.pone.0167323

**Published:** 2016-11-30

**Authors:** Jeanine Schibler, Ann M. Tomanek-Chalkley, Jessica L. Reedy, Fenghuang Zhan, Douglas R. Spitz, Michael K. Schultz, Apollina Goel

**Affiliations:** 1 Interdisciplinary Graduate Program in Molecular and Cellular Biology, University of Iowa, Iowa City, IA, United States of America; 2 Free Radical and Radiation Biology Program, Department of Radiation Oncology, University of Iowa, Iowa City, IA, United States of America; 3 Department of Internal Medicine, University of Iowa, Iowa City, IA, United States of America; 4 Department of Radiology, University of Iowa, Iowa City, IA, United States of America; Instituto Nacional de Cardiologia, MEXICO

## Abstract

Therapeutic advances have markedly prolonged overall survival in multiple myeloma (MM) but the disease currently remains incurable. In a panel of MM cell lines (MM.1S, OPM-2, H929, and U266), using CD138 immunophenotyping, side population staining, and stem cell-related gene expression, we demonstrate the presence of stem-like tumor cells. Hypoxic culture conditions further increased CD138^low^ stem-like cells with upregulated expression of *OCT4* and *NANOG*. Compared to MM cells, these stem-like cells maintained lower steady-state pro-oxidant levels with increased uptake of the fluorescent deoxyglucose analog. In primary human MM samples, increased glycolytic gene expression correlated with poorer overall and event-free survival outcomes. Notably, stem-like cells showed increased mitochondrial mass, rhodamine 123 accumulation, and orthodox mitochondrial configuration while more condensed mitochondria were noted in the CD138^high^ cells. Glycolytic inhibitor 2-deoxyglucose (2-DG) induced ER stress as detected by qPCR (*BiP*, *ATF4*) and immunoblotting (BiP, CHOP) and increased dihydroethidium probe oxidation both CD138^low^ and CD138^high^ cells. Treatment with a mitochondrial-targeting agent decyl-triphenylphosphonium (10-TPP) increased intracellular steady-state pro-oxidant levels in stem-like and mature MM cells. Furthermore, 10-TPP mediated increases in mitochondrial oxidant production were suppressed by ectopic expression of manganese superoxide dismutase. Relative to 2-DG or 10-TPP alone, 2-DG plus 10-TPP combination showed increased caspase 3 activation in MM cells with minimal toxicity to the normal hematopoietic progenitor cells. Notably, treatment with polyethylene glycol conjugated catalase significantly reduced 2-DG and/or 10-TPP-induced apoptosis of MM cells. Also, the combination of 2-DG with 10-TPP decreased clonogenic survival of MM cells. Taken together, this study provides a novel strategy of metabolic oxidative stress-induced cytotoxicity of MM cells via 2-DG and 10-TPP combination therapy.

## Introduction

Multiple myeloma (MM) is a debilitating plasma cell malignancy. The American Cancer Society estimates that in the United States in 2016 approximately 30,000 new cancer diagnosis and 12,500 deaths will be linked with MM [1]. New pharmacological drugs (i.e. lenalidomide, pomalidomide, bortezomib, carfilzomib, ixazomib, panobinostat) show anti-MM activity and in combination with conventional therapies offer deep clinical remissions [2]. However, most patients eventually relapse with aggressive drug-resistant MM disease [3]. Resistance to chemotherapy and MM relapse has been attributed to persistence of residual disease that contains drug-resistant phenotypes i.e. de-differentiated pre-plasma cells, genetically distinct clones, and stem-like tumor cells [4–6]. Although the cancer stem cell (CSC) hypothesis remains controversial in MM, several groups have demonstrated a distinct stem-like sub-population that displays increased clonogenicity, sustained self-renewal, differentiation towards CD138^+^ mature MM cells, and chemo-resistance [5, 7, 8]. Therefore, successful therapy requires an approach to induce cytotoxicity in the drug-resistant tumor cells with the overall objective to reduce MM recurrence.

Cancer cells almost universally show metabolic reprogramming with an increased reliance on aerobic glycolysis (Warburg effect) [9]. In cancer cells, an increased glycolytic flux is believed to provide biosynthetic intermediates for cell proliferation and also compensate for upregulated reactive oxygen species (ROS i.e. O_2_^•−^ and H_2_O_2_) formed as by-product of aberrant electron transport chain (ETC) activity [10–13]. Glucose metabolism generates pyruvate that can directly detoxify peroxides and NADPH that act as reducing equivalents in thioredoxin and glutathione systems [13–15]. Recent studies have shown that targeting the glycolytic pathway results in cytotoxicity in MM cells [16–20]. Similar to cancer cells, adult stem cells and CSCs have been reported to show metabolic reprogramming with altered redox homeostasis [21, 22]. Notably, CSCs can exhibit dependence on glycolytic metabolism or mitochondrial oxidative phosphorylation (OXPHOS) that is integral to their survival and self-renewal properties [23–26]. To the best of our knowledge, the pro-oxidant metabolic state of MM stem-like cells has not been deciphered or used as a target for inhibition of MM disease recurrence.

Studies have shown that targeting oxidative metabolism can provide a therapeutic benefit in cancer [12, 27–29]. Our published results have established a biochemical rationale (based on intrinsic differences in cancer vs. normal cell metabolism) for manipulating ROS to augment MM cytotoxicity [30, 31]. We have also established isogenic MM cell lines with acquired bortezomib resistance and published that bortezomib resistance correlates with upregulated expression of antioxidant enzymes [32]. In the present study, we compromised cellular antioxidant function and increased pro-oxidant production with the aim to selectively induce oxidative stress-mediated cytotoxicity in the MM stem-like fractions. For inhibition of glycolytic and oxidative metabolism, we utilized 2-deoxyglucose (2-DG), which is being actively pursued in clinical trials as an adjuvant to cancer chemotherapy [33, 34]. Biochemically, hexokinase converts 2-DG to 2-deoxy-glucose-6-phosphate (2-DG-6-P), an intermediate that cannot be metabolized by phosphoglucose isomerase resulting in the downstream inhibition of glycolysis and induction of oxidative stress [12, 35]. Also, besides inhibiting glycolysis, 2-DG can interfere with N-linked protein glycosylation, induce endoplasmic reticulum (ER) stress, and apoptosis [36].

Similar to cancer cells, CSCs also show disruptions in mitochondrial ETC function, making them vulnerable to approaches that further increase mitochondrial pro-oxidant production or compromise cellular antioxidant function [28, 37]. To enhance cellular ROS levels, we utilized a triphenylphosphonium derivative, decyl-TPP (10-TPP) [38, 39]. Studies show that TPP^+^ derivatives accumulate in mitochondria, driven by the mitochondrial membrane potential (Δψ_m_), and induce cytotoxicity in cancer cells [40–42]. Furthermore, CSCs can exhibit altered Δψ_m_ and therefore show increased accumulation of TPP derivatives and cytotoxicity [24, 43–45].

In this study, we have characterized the oxidative metabolic aberrations of MM stem-like cells and show that compared to bulk tumor cells, these stem-like cells maintain lower steady-state pro-oxidant levels with increased glucose uptake. We show that by combining 2-DG with 10-TPP, we can effectively reduce clonogenic fractions in MM cell lines without increasing cytotoxicity towards normal hematopoietic progenitor cells. The proposed first-in-class combination treatment of 2-DG with 10-TPP provides a novel way to induce cytotoxicity in tumor cells and can potentially inhibit chemo-resistance that is universally and rapidly encountered in MM therapy.

## Materials and Methods

### Ethics statement

All procedures involving animals in this study were carried out in accordance with the recommendations set forth in the Guide for the Care and Use of Laboratory Animals of the National Institutes of Health. The protocol was reviewed and approved by the Institutional Animal Care and Use Committee (IACUC), University of Iowa, Iowa (Protocol Number 1312229).

### Cell culture

Human MM cell lines (HMCLs) MM.1S (CRL-2974), U266 (TIB-196), H929 (CRL-9068), and RPMI8226 (CCL-155) were purchased from American Type Culture Collection (ATCC, Manassas, VA) while OPM-2 cell line was obtained from Dr. Shaji Kumar (Mayo Clinic, Rochester, MN). All cell lines were cultured in complete medium consisting of RPMI 1640 (Thermo Fisher Scientific, Waltham, MA), 10% fetal bovine serum (FBS, Gibco, Thermo Fisher Scientific), 100 mg/mL streptomycin and 100 U/mL penicillin (Thermo Fisher Scientific), and 50 μM β-mercaptoethanol at 37°C under 21% O_2_ (normoxia) [31]. For experiments performed under hypoxic conditions, cells were placed in a modular incubator chamber (Billups-Rothenberg, Del Mar, CA), flushed with 1% O_2_ balanced with N_2_, sealed, and cultured at 37°C in the regular CO_2_ incubator.

The normal hematopoietic progenitor cells (HPCs) were enriched from C57BL/6 mice (9–11 weeks old, The Jackson Laboratory, Bar Harbor, ME) as previously described [30]. All animal work was performed in accordance with the guidelines of the Institutional Animal Care and Use Committee (IACUC) of the University of Iowa (protocol Number 1312229). Mice were sacrificed by euthanasia with CO_2_ gas inhalation followed by cervical dislocation. Bone marrow was harvested and depleted for lineage negative cells using the EasySep™ Mouse HPC Enrichment Kit (#19756, Stem Cell Technologies, Vancouver, BC, Canada). Cell population was confirmed using APC-conjugated c-Kit and PE-conjugated Sca-1 antibodies (#17–1171, #12–5981, eBioscience, San Diego, CA).

### Flow cytometric analysis of stem-like MM fractions

For CD138 immunophenotyping, cells were plated at 1.0 x 10^6^/mL for 24 h in RPMI complete medium. Labeling was done with APC- or FITC-conjugated CD138 antibody (#17–1389 or #11–1389, eBioscience) for 30 min on ice. Hoechst 33258 staining (5 μg/mL Sigma-Aldrich, St. Louis, MO) was used to exclude dead cells. Sequential gating for viability, forward and side scatter was done. The CD138^low^ cells were gated by comparing the treatment samples to the unstained as well as the isotype control. The gate was drawn at the natural breakpoint between the populations, around 10^3^ on the Y-axis. The same gate was applied to all samples within a cell line. CD138^low^ cells (a minimum of 3000 events) were captured on a BD LSR-Violet or LSR-UV (Flow Cytometry Core, UI) using λ_ex_ = 633 nm and λ_em_ = 660/20 nm bandpass filter (APC), λ_ex_ = 488 nm or λ_em_ = 530/30 nm bandpass filter (FITC). Data acquisition and analysis was performed with FlowJo software (Ashland, OR) and the median fluorescent intensity (MFI) of the CD138^low^ and CD138^high^ population was calculated and corrected using unlabeled cells. The MFI for the CD138^high^ population was averaged and used to calculate the fold change between the populations.

SP staining was performed as previous described [46]. Briefly, cells were stained at 37°C with Hoechst 33342 (5 μg/mL, Sigma-Aldrich) in pre-warmed complete media for 90 minutes. The ABC transport pump inhibitor verapamil (100 μM, Sigma-Aldrich), which inhibits the dye efflux, was added with Hoechst 33342 and used as a control. Cells were pelleted at 4°C, re-suspended in cold Hank’s Balanced Saline Solution (HBSS, Thermo Fisher Scientific) containing 2% FBS, and kept on ice prior to analysis. A minimum of 200,000 events were captured on a BD-FACS Aria II flow cytometer (Becton Dickinson) using λ_ex_ = 355 nm, λ_em_ = 450/50 nm bandpass filter (Hoechst blue) and λ_em_ = 675/50 nm bandpass filter (Hoechst red). Flow cytometric data acquisition and analysis was performed with DIVA software (Becton Dickinson). SP cells were identified by comparing to the verapamil treated samples.

### Measurements of intracellular pro-oxidant levels

Oxidation-sensitive probes carboxy-2’,7’-dichlorodihydrofluorescein diacetate (carboxy-H_2_DCF-DA, Sigma-Aldrich), dihydroethidium (DHE, Sigma-Aldrich) or peroxy orange-1 (PO-1, Tocris Bioscience, Avonmouth, Bristol, UK) were used. In brief, cells were plated for 24 h at 1.0 x 10^6^/mL in RPMI complete medium. For measuring 10-TPP mediated increases in dye oxidation, cells were treated with 10-TPP (Alfa Aesar, Ward Hill, MA) for 1.5 h (1 μM, MM.1S or 2.5 μM, OPM-2). Labeling with H_2_DCF-DA (10 μg/mL, 15 min at 37°C for hydroperoxide measurement) in PBS or DHE (10 μg/mL, 40 min at 37°C for superoxide measurement) in PBS + 5% sodium pyruvate was done as previously described by us [31]. PO-1 labeling (10 μg/mL, 1.5 h at 37°C for peroxide measurement) was done in complete media [13]. CD138 and H_2_DCF-DA/DHE co-staining was done by placing cells on ice and adding APC-CD138 for 30 min. SP and PO-1 co-staining was achieved by simultaneously adding Hoechst 33342 (5 μg/mL) and PO-1 dyes. Cells were pretreated with menadione (100 μM; 1.5 h at 37°C, Sigma-Aldrich) or H_2_O_2_ (250 μM, 1.5 h, Sigma-Aldrich) as a positive control for H_2_DCF-DA and PO-1 oxidation. Antimycin A (10 μM, Sigma-Aldrich) was added with DHE staining as a positive control. Samples were analyzed by flow cytometry using λ_ex_ = 488 nm and λ_em_ = 530/30 nm bandpass filter (H_2_DCF-DA), λ_ex_ = 488 nm and λ_em_ = 610/20 nm bandpass filter (DHE), or λ_ex_ = 561 nm and λ_em_ = 582/15 nm bandpass filter (PO-1).

Mitochondrial oxidant production was measured by MitoSOX Red staining (5 mM for 15 min at RT, Invitrogen, Thermo Fisher Scientific) after 10-TPP treatment (1 or 2.5 μM, for MM.1S and OPM-2, respectively) [31]. The specificity of MitoSOX staining was confirmed using cells transduced with adenovirus expressing MnSOD (Ad-MnSOD) or control (Ad-CMV) as described by us [31]. Antimycin A (100 μM for 2 h) was used as positive control. These were then washed, placed on ice, and stained with APC-CD138 antibody for 30 min. Samples were analyzed by flow cytometry using λ_ex_ = 488 nm and λ_em_ = 585/42 nm bandpass filter (MitoSOX).

### Fluorescent glucose uptake assay

Cells were plated at 3.5 x 10^5^ cells/mL in RPMI complete medium for 3 days under normoxia or hypoxia. Cells were labeled with 2-(*N*-(7-nitrobenz-2-oxa-1,3-diazol-4-yl) amino)-2-deoxyglucose (2-NBDG, Thermo Fisher Scientific) (20 μM) for 2 h at 37°C [47]. These were then washed, placed on ice and stained with APC-CD138 for 30 min. Samples were analyzed by flow cytometry using λ_ex_ = 488 nm and λ_em_ = 530/30 nm bandpass filter (2-NBDG).

### Assessment of mitochondrial mass

Cells were plated at 1 x 10^6^ cells/mL in RPMI complete medium for 24 h followed by labeling with MitoTracker Green (Molecular Probes, Thermo Fisher Scientific, 0.1 μM) for 30 min at 37°C. Cells were washed, placed on ice, and stained with APC-CD138 antibody for 30 min. Samples were analyzed by flow cytometry using λ_ex_ = 488 nm and λ_em_ = 530/30 nm bandpass filter (MitoTracker Green).

### Quantitative real-time PCR (qPCR) and RT-PCR

Total RNA was isolated using the Direct-zol RNA kit (R2050, Zymo, Irvine, CA) and quantified on a NanoDrop 1000. cDNA was synthesized from 1 μg of total RNA, using the iScript cDNA synthesis kit (#170–8891, Bio-Rad Laboratories, Hercules, CA) as described by us previously [31]. The cDNAs were subjected to qPCR analysis with VEGF primers (5′→3′, CCTTGCTGCTCTACCTCCAC and CCACTTCGTGATGATTCTGC forward and reverse, respectively, amplicon length of 54 bp), BiP primers (5′→3′, TGTTCAACCAATTATCAGCAAACTC and TTCTGCTGTATCCTCTTCACCAGT forward and reverse, respectively, amplicon length of 73 bp), ATF4 primers (5′→3′, GTTCTCCAGCGACAAGGCTA and ATCCTCCTTGCTGTTGTTGG forward and reverse, respectively, amplicon length of 88 bp), or primers listed in Table 1. Primers design was done utilizing the Universal Probe Library Assay Design Center (Roche, Indianapolis, IN) specifying for intron spanning regions for the respective genes. qPCR was performed in a 10 μL reaction using the synthesized cDNA (10 ng), forward and reverse primers (100 μM), and 0.75x FastStart Universal SYBR Green Master (#04913850001, Roche). The assay was plated in triplicate and run on an ABI-7500 Fast and analyzed using the ABI 7500 software v2.3. The C_T_ values for the target genes in all of the samples were normalized to the 18S transcript, and the fold difference (relative abundance) was calculated using the formula 2^-ΔΔ^C_T_ and mean value was plotted.

**Table 1:** qPCR primers and fold changes in mRNA expression ± SEM of various genes.

Gene	Primer sequence (5’→3’)	Size (bp)	Cell line	Cell type	Fold change
**OCT4**	For -CCTGTCTCCGTCACCACTCT Rev—GGCACAAACTCCAGGTTTTC	78	MM.1S MM.1S OPM-2 OPM-2 H929 U266	CD138^low^ vs. CD138^high^ SP vs. MP CD138^low^ vs. CD138^high^ SP vs. MP CD138^low^ vs. CD138^high^ CD138^low^ vs. CD138^high^	3.0 vs. 1.2 1.3 vs. 1.0* 182.5 vs. 2.9 4.8 vs. 1.2 2.0 vs. 0.9* 71.6 vs. 3.3
**NANOG**	For—TGTGGGCCTGAAGAAAACTAT Rev—GCTGTCCTGAATAAGCAGATC	67	MM.1S OPM-2 H929 U266	CD138^low^ vs. CD138^high^ CD138^low^ vs. CD138^high^ CD138^low^ vs. CD138^high^ CD138^low^ vs. CD138^high^	2.5 vs. 0.7 138.8 vs. 1.9 1.7 vs. 1.2* 28.4 vs. 2.5
**BCRP**	For—TGGCTTAGACTCAAGCACAGRev—TCGTCCCTGCTTAG ACATCC	67	MM.1S OPM-2 H929 U266	CD138^low^ vs. CD138^high^ CD138^low^ vs. CD138^high^ CD138^low^ vs. CD138^high^ CD138^low^ vs. CD138^high^	1.0 vs. 0.7* 98.4 vs. 13.1 2.1 vs. 1.7 42.0 vs. 13.0
**SDC1**	For—TCTGACAACTTCTCCGGCTC Rev—CCACTTCTGGCAGGACTACA	171	MM.1S MM.1S OPM-2 OPM-2 H929 U266	CD138^low^ vs. CD138^high^ SP vs. MP CD138^low^ vs. CD138^high^ SP vs. MP CD138^low^ vs. CD138^high^ CD138^low^ vs. CD138^high^	0.5 vs. 2.6 1.1 vs. 1.0* 0.4 vs. 1.5* 21.6 vs. 2.5 8.3 vs. 12.2 1.0 vs. 3.2

Cell preparations from the same parental population were separated by flow cytometry and examined in parallel. Technical triplicates of two independent sorts for CD138 expression and side population were examined.

* Data is normalized to 18S and fold change is calculated by normalization to the lowest mRNA expression for each cell type (indicated by*).

### Microarray analysis of glycolytic gene expression and clinical prognosis

The gene expression profiling data of MM Total Therapy 2 (TT2) study [48] was analyzed for transcriptional expression of glycolytic genes. Principal components analysis (PCA) was performed to create a genetic signature for glycolytic genes. We used GEP data for *ALDOA* (Affymetrix ID 200966, 214687, 238996), *TPI1* (Affymetrix ID 200822, 210050, 213011), *GAPDH* (Affymetrix ID 212581, 213453, 217398), *PGK1* (Affymetrix ID 200737, 200738, 217356), *PKM* (Affymetrix ID 2201251), and *LDHA* (Affymetrix ID 200650). In the first step, PCA was applied to genes with more than one probe site to create a unique gene-specific variable. In the second step, PCA was applied to all the gene-specific variables to create a universal genetic signature. In each step, the first principal component was retained. Using Cox proportional hazards regression, overall survival (OS) and event-free survival (EFS) were compared between Q1 and Q2+Q3+Q4 of the genetic signature. Plots of the Kaplan-Meier estimated cumulative probabilities of OS and EFS were constructed (Biostatistics Core, UI).

### Western blotting

Cells (HMCLs or HSCs) were plated at 1 x 10^6^/mL in RPMI complete medium overnight and then treated for 24 h with 2-DG (20 mM) and/or mannose (Sigma-Aldrich, 20 mM) and/or10-TPP (0.5 μM). Cells were collected, washed with cold PBS, and lysed in radioimmunoprecipitation assay buffer with protease inhibitors (Roche, Indianapolis, IN). Protein concentration was estimated using Bradford reagent (Bio-Rad Laboratories, Hercules, CA). Equal protein amounts were electrophoresed on a 4–15% gradient gel (Bio-Rad Laboratories). Proteins were transferred using the semi-dry method to a PVDF membrane and blocked in 5% non-fat milk in TBST (4 mM Tris base pH 7.5, 10 mM NaCl, 0.1% Tween-20). Blots were incubated with primary antibody overnight at 4°C, washed, and incubated with species-specific horseradish peroxidase-conjugated secondary antibody. Caspase-3 antibody (1:1000 dilution, #9662, Cell Signaling Technology, Danvers, MA) and MnSOD antibody (1:500 dilution, #AF3419, R&D Systems, Minneapolis, MN) were used. For ER stress analysis, antibodies against BiP (1:500 dilution, #3177, Cell signaling) or CHOP (1:250 dilution, #2895, Cell signaling) were used. β-actin was used at 1:1000 dilution (JLA20, Developmental Studies Hybridoma Bank, UI)[49]. Blots were developed with Pierce ECL Plus (Thermo Fisher Scientific) and imaged on a Typhoon FLA 7000 (GE Healthcare Bio-Sciences, Pittsburg, PA). Protein expression was quantified using ImageJ software.

### Measurement of Δψ_m_ by rhodamine (Rh)123

Cells were plated at 1 x 10^6^ cells/mL in RPMI complete medium for 24 h. Samples were labeled with Rh123 (#R8004, Sigma-Aldrich, 10 μg/mL) for 15 min at 37°C, washed, and MFI was measured by flow cytometry using λ_ex_ = 488 nm and λ_em_ = 530/30 nm bandpass filter (Rh123) [50].

### Assessment of apoptosis by annexin V-FITC and PI assay

MM.1S or OPM-2 cells (1 x 10^6^/mL) were seeded in RPMI complete medium and incubated overnight. These were then treated with 2-DG (20 mM) and/or 10-TPP (0.5 μM) for 12 h; specific wells were pretreated with PEG-catalase (100 U/mL for 1 h, Sigma-Aldrich)[30] before and during 2-DG and/or 10-TPP treatment. Apoptosis was detected by annexin V FITC and PI staining (Cayman Chemical, Ann Arbor, Michigan) and flow cytometry analysis [31, 51].

### Clonogenic survival assay

To determine clonogenic potential of unsorted HMCLs, *in vitro* limiting dilution assay was done as published by us [32]. Cells were plated overnight at 2.5 x 10^5^/mL in RPMI complete medium and treated for 24 h with 10-TPP [0.02 or 0.1 μM (for MM.1S) and 0.2 or 1 μM (for OPM-2)] and/or 2-DG (20 mM). Cells were then plated in a U bottom 96-well plate, cultured for 10 days, and scored. The plating efficiency (PE), survival fractions, and normalized survival fraction (NSF) was calculated for each treatment.

### Confocal imaging of 10-TPVP

Cells were plated at 1 x 10^6^/mL RPMI complete medium for 24 h. Mitochondrial imaging was using 10-TPVP, kindly provided by from the Pigge lab (Dr. F. C. Pigge, Division of Organic Chemistry, University of Iowa, IA) [52]. In brief, cells were incubated with 10-TPVP (1 μM for 1.5 h) at 37°C [53], washed in PBS, and stained with MitoTracker Red CM-H_2_XRos (Invitrogen, 0.1 μM for 30 min) at 37°C. Cells were re-suspended in 0.1 mL ice cold PBS and stored on ice in dark. For live imaging, cells were mounted in PBS and images were obtained using a Confocal Laser Scan Microscope (Leica SP8 3x STED system, Germany) at the Central Microscopy Research Facility, UI. CCCP (5 μM for 2 h was used as negative control. 10-TPVP λ_ex_ = 330−385 nm, λ_em_ = 449–520 nm. For improving the quality of 10-TPVP image as well as the co-localization image of 10-TPVP with mitoTracker red, 10-TPVP fluorescence images, post-acquisition, were pseudo-colored from blue to green using Adobe software.

### Electron microscopy and mitochondrial configuration analysis

HMCLs were enriched for CD138^low^ and CD138^high^ cells and visible pellets (containing approximately 10^6^ cells) were fixed overnight in 20 volumes of 2.5% gluteraldehyde in 0.1 M sodium cacodylate buffer. Post-fixation was done for 30 min at room temperature with a buffered 1% osmium tetroxide solution reduced with 1.5% potassium ferrocyanide. Samples were stained with 2.5% uranyl acetate and then rinsed and fully dehydrated using increasing concentrations of ethanol. Infiltration of Eponate 812 epoxy resin and ethanol was carried out over several hours to 100% resin and cured overnight in a 60°C oven. Sections of 80 nm thickness were cut using a Leica UC6 Ultramicrotome II. Grids were then counterstained with 5% uranyl acetate for 3 min and Reynold's lead citrate for 2 min. Samples were imaged using a JEOL JEM-1230 transmission electron microscope at 120 kV (NIH grant 1 S10 RR018998-01, Central Microscopy Research Facility, UI). To perform ultrastructure analysis, all mitochondria were selected and a minimum of approximately 20 mitochondria per cell type were manually scored by 7 unbiased volunteers and assigned to one of two broad categories: condensed (mitochondrial density > cytoplasmic density) or orthodox (mitochondrial density = cytoplasmic density) [54, 55]. The ratio of orthodox/condensed mitochondria was calculated for each sample.

### Statistical analysis

GraphPad Prism 6.0 software (San Diego, CA) was used for data handling, analysis, and presentation. For all experiments, data depicts average of repeat experiments (n = 2 or 3) run in technical replicates (n = 3) as indicated in figure legends. Statistical significance was determined using either a 2-way ANOVA or two-tailed unpaired *t* test with a confidence interval of 95%. Microarray data analysis was done using R (http://www.r-project.org). For Kaplan–Meier survival curves, all statistical tests were two-sided and assessed for significance with the SAS 9.3 software package (Cary, NC). P < 0.05 was considered to be statistically significant.

## Results

### HMCLs contain a distinct stem-like tumor cell population

**Fig 1:**
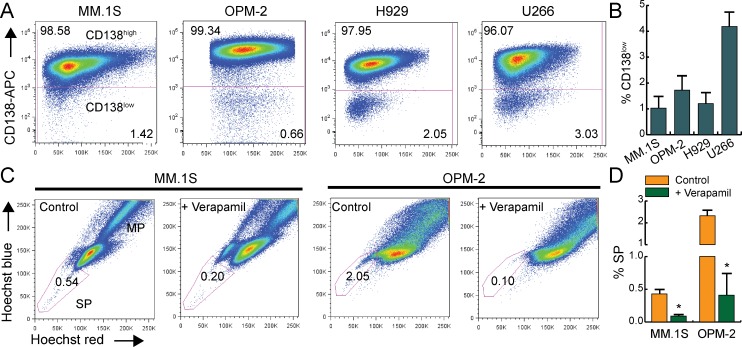
Flow cytometric analysis of stem-like cells in HMCLs. A. Representative dot plots for CD138 vs. side scatter and B. quantification of % CD138^low^ fractions. C. Hoechst 33342 staining for SP with or without verapamil. Gate represents the % SP fractions, MP = main population. D. Quantification of % SP cells in MM.1S and OPM-2 cell lines ± verapamil. Bars represent mean of three independent runs ± SEM, *p < 0.05 vs. control.

Low expression of CD138 (syndecan-1, plasma cell differentiation marker) has been used as a phenotypic marker for MM stem-like cells [56–58]. In MM.1S, OPM-2, H929, and U266 cells, a distinct sub-population of CD138^low^ cells (approximately 1–4.2%) was detected by flow cytometry (Fig 1A and 1B). As the robustness of CD138 to correctly identify stem-like MM cells remains debatable [59], side population (SP) staining was also done which an established functional marker of adult stem cells and many types of CSCs including MM [6, 60–62]. The SP fractions in MM.1S and OPM-2 cells were ~ 0.4% ± 0.1% and 2.3% ± 0.5%, respectively and verapamil treatment reduced SP fraction by approximately 80% confirming correct SP gating (Fig 1C and 1D).

Gene expression profiling has shown a higher expression of induced pluripotent stem cell genes in stem-like CD138^-^ than bulk CD138^+^ cells isolated from HMCLs [63]. We enriched the CD138^low^ and CD138^high^ cells from our HMCL panel and examined mRNA expression of stem cells transcription factor genes (OCT4, NANOG), ATP-binding cassette (ABC) transporter family member ABCG2/ breast cancer resistance protein (BCRP), and syndecan-1 (SDC1) by qPCR. For all HMCLs, CD138^low^ cells showed higher *OCT4*, *NANOG* and *BCRP* expression relative to the CD138^high^ cells (Table 1). A low *SDC1* expression confirmed that CD138^low^ cells were genetically distinct and not comprised of MM cells that had shed surface CD138 (Table 1) [64]. To assess if SP staining may better represent stem-like MM cells, SP and main population (MP) cells were enriched from MM.1S and OPM-2 cells. For both cell lines, SP cells showed higher *OCT4* expression than MP cells (Table 1). Notably, SP cells have been reported to be CD138^-/low^ in clinical MM samples [65]. Furthermore, CD138^low^ cells were detected in MM patients [56, 66] and a retrospective study correlated poor prognosis with increased CD138^low^ cells at diagnosis and relapse [67]. Based on these published results and our findings (Fig 1 and Table 1), we postulate that CD138 immunostaining is a useful way to identify stem-like cells in HMCLs. For subsequent experiments we have primarily utilized CD138 surface expression to identify stem-like MM cells.

### Hypoxia increases CD138^low^ cell population in HMCLs

**Fig 2:**
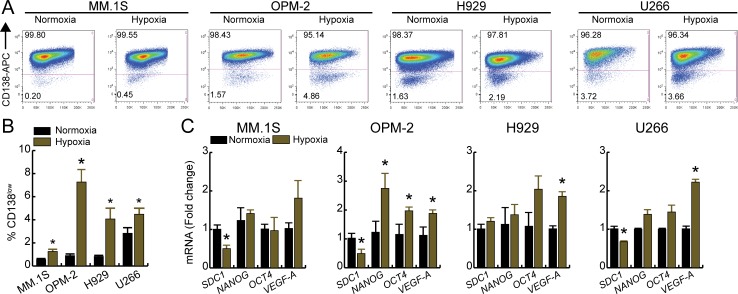
Hypoxia increases CD138^low^ population and alters transcriptional profile of HMCLs. Cell were cultured at either normoxia (21% O_2_) or hypoxia (1% O_2_) for 3 days, labeled with CD138-APC antibody and the percentage of CD138^low^ and CD138^high^ cells were analyzed by flow cytometry. A. Representative dot plots of different HMCLs and B. quantification of % CD138^low^ fractions under normoxia or hypoxia. C. qRT-PCR analysis of *SDC1*, stem cell genes (*NANOG*, *OCT4*), and *VEGF-A*. For panels B and C, bars represent mean of three independent runs ± SEM. *p < 0.05 vs. normoxia.

Studies show that culturing HMCLs under short-term hypoxia (2–3 days) reduces CD138 mRNA and protein expression [68, 69]. Also, long-term culturing under hypoxia (30 days) was reported to increase protein and mRNA expression of OCT4, NANOG and SOX2 [68]. Wen *et al*. [65] reported a 5-fold increase in SP cells (8226 and U266 cells) when cultured under hypoxia for 5 days that was suggested to arise from MP cells without any additional stimuli. To assess hypoxia-mediated alterations in the number CD138^low^ cells, we cultured HMCLs in 1% O_2_ for 3 days. CD138 immunophenotyping showed that hypoxia significantly increased the %CD138^low^ cells compared to the normoxic cultures (Fig 2A) with a 2.2, 8.1, 5.0, and 1.6-fold increase for MM.1S, OPM-2, H929, and U266 cells, respectively (Fig 2B).

To verify hypoxia-mediated increases in stem-like cell population, we analyzed *SDC1*, *OCT4*, and *NANOG* expression using unsorted normoxic and hypoxic cells; VEGF-A is a well-established HIF-1 regulated gene and was included as an indicator of hypoxia. For OPM-2 cells, hypoxia significantly increased *OCT4*, *NANOG* while *SDC1* expression decreased presumably due to increase in the stem-like cells (Fig 2C). For other HMCLs, a trend towards increased expression of *OCT4* and/or *NANOG* and decreased expression of *SDC1* was noted with hypoxia (Fig 2C). Overall, Fig 2 shows that hypoxia increases CD138^low^ population and alters stem cell gene expression levels in MM cells. We have used low pO_2_ levels to further characterize oxidative metabolism of stem-like MM cells.

### MM stem-like cells maintain lower steady-state pro-oxidant levels

**Fig 3:**
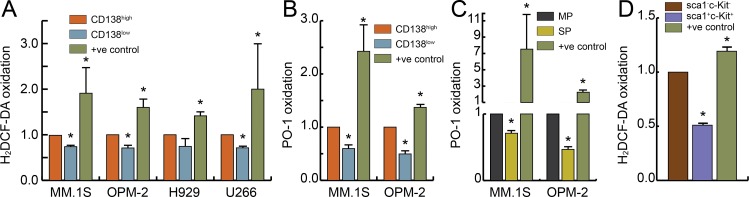
MM stem-like cells and HPCs maintain lower steady-state ROS levels. HMCLs were stained with APC-CD138 antibody and A. H_2_DCF-DA or B. PO-1 oxidation was measured in CD138^low^ and CD138^high^ cells by flow cytometry. Results are presented as the fold change relative to CD138^high^ cells (set to 1). C. MM.1S or OPM-2 cells were stained with Hoechst 33342 and PO-1 oxidation was determined by flow cytometry in SP and MP cells. Results are presented as the fold change relative to MP cells (set to 1). D. Murine HPCs were enriched and H_2_DCF-DA oxidation was compared between sca1^-^c-Kit^-^ (set to 1) and sca1^+^c-Kit^+^ cells. As a positive control, menadione or H_2_O_2_ treatment was used. Bars represent mean of three independent runs ± SEM. *p < 0.01 vs. CD138^high^/ MP/ sca1^-^c-Kit^-^ cells.

We used the H_2_DCF-DA probe to determine endogenous steady-state pro-oxidants levels in CD138^high^ and CD138^low^ cells in the HMCLs. In MM.1S and OPM-2 cell lines, CD138^low^ cells showed a significant lower H_2_DCF-DA probe oxidation (~25–30%) relative to the CD138^high^ cells (Fig 3A). A similar trend of CD138^low^ cells maintaining lower steady-state pro-oxidants compared to the bulk CD138^high^ was noted for H929 and U266 cell lines (Fig 3A). We used the more specific oxidation sensitive PO-1 boronate probe and detected lower H_2_O_2_ levels (~40–50%) in CD138^low^ compared to CD138^high^ cells for MM.1S and OPM-2 cells (Fig 3B). Notably, in MM.1S and OPM-2 cells, the SP cells also showed reduced PO-1 oxidation compared to the MP cells (Fig 3C) substantiating the novel observation that MM stem-like cells maintain lower steady-state pro-oxidant levels compared to bulk MM cells.

Next, we used mouse bone marrow to obtain HPCs with ~2.4 enrichment in the sca1^+^c-Kit^+^ cells (data not shown). By H_2_DCF-DA probe labeling, sca1^+^c-Kit^+^ cells showed approximately 50% lower probe oxidation relative to the sca1^-^c-Kit^-^ cells (Fig 3D). Overall, Fig 3 results show that MM stem-like cells and sca1^+^c-Kit^+^ HPCs maintain lower steady-state pro-oxidant levels compared to their differentiated counterparts (bulk MM or sca1^-^c-Kit^-^ bone marrow cells, respectively). Therefore, a pharmacological approach that selectively induces oxidative stress-mediated killing of MM stem-like cells with minimal toxicity to the normal HPCs is needed to gain a therapeutic benefit in MM.

### Poor MM prognosis associates with increased glycolytic gene expression

**Fig 4:**
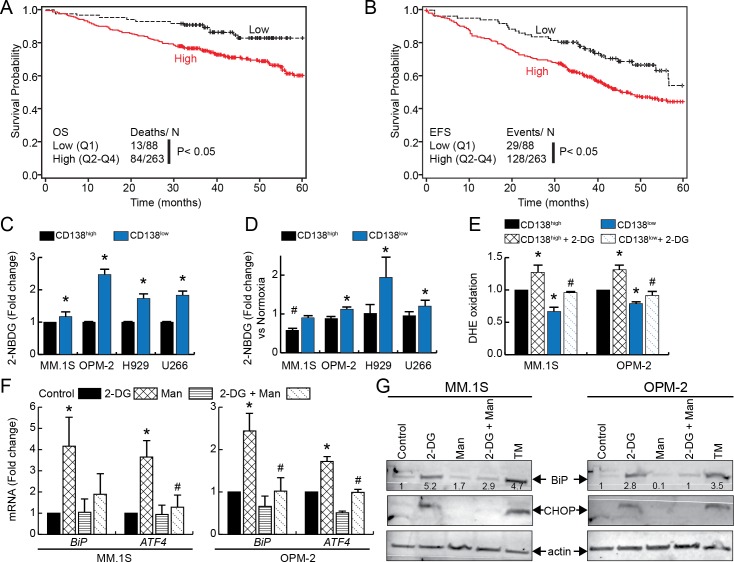
Glycolytic gene expression correlates with MM patient survival and increased glucose uptake can induce 2-DG-mediated oxidative and ER stress in of CD138^low^ cells. Kaplan–Meier graphs from TT2 trial clustered on glycolytic gene signatures (*HK2*, *ALDOA*, *TPI1*, *GAPDH*, *PGK1*, *PKM2*, *and LDHA*) showing cumulative probabilities of A. OS and B. EFS in MM patients. Glucose uptake assays in HMCLs cultured under C. normoxia or D. hypoxia for 3 days. Cells were incubated with 2-NBDG followed by APC-CD138 staining and flow analysis. For panel C, mean fluorescence values (MFI) values was normalized to CD138^high^ cells and presented fold change. *p < 0.01 vs. control. Bars represent mean of three independent runs ± SEM. For panel D, 2-NBDG uptake under hypoxia was compared with normoxia for CD138^low^ and CD138^high^ separately and depicted as hypoxia-induced fold change. *p < 0.01 vs. CD138^high^ cells under normoxia, ^#^p < 0.01 vs. CD138^low^ cells under normoxia. E. MM.1S and OPM-2 cells were treated without or with 2-DG for 1.5 h followed by DHE staining and flow analysis. Normalized MFI relative to CD138^high^ cells are shown. MM.1S and OPM-2 cells were treated with 2-DG and/or mannose or 24 h followed by F. qRT-PCR analysis of *BiP* or *ATF4* G. Western blot analysis for BiP (78 kDa), CHOP (27 kDa) or β-actin (42 kDa, loading control), tunicamycin (TM, 5 μM is used as a positive control. The quantification of BiP after normalization to untreated control is shown below each band. For panels D-F, bars represent mean of three independent runs ± SEM; *p < 0.01 vs. CD138^high^ or control untreated cells, ^#^p < 0.01 vs. CD138^low^ or 2-DG treated cells.

Clinically, relapse and dismal MM prognosis are associated with elevated glycolytic flux [70–72]. To extend this observation, we determined if increased expression of glycolytic genes correlates with clinical outcomes in MM. We queried the microarray dataset and associated clinical information from the TT2 study [73]. Gene signature associated with glycolysis (hexokinase II, aldolase A, triosephosphate isomerase I, glyceraldehyde-3-phosphate dehydrogenase, phosphoglycerate kinase I, pyruvate kinase M2, lactate dehydrogenase A) was used. A significant difference between the lower and upper quartile of glycolytic signature for both OS (Fig 4A) and EFS was seen (Fig 4B). The risk of death for patients with a high glycolytic gene expression was 2.9X that with a low gene expression (95% CI, 1.5–5.5). Additionally, the risk of an event for individuals in the high group was 1.9X that of the low group (95% CI, 1.2–3.0). Therefore, increased glycolytic gene expression appears to serve as an independent predictor of MM patient mortality and targeting glycolytic metabolism could potentially improve survival outcome in MM.

### Increased glucose uptake and 2-DG mediated ER and oxidative stress in MM cells

To determine if CD138^low^ stem-like cells display increased glucose uptake, HMCLs were stained with 2-NBDG and CD138-APC and analyzed by flow cytometry. Compared to CD138^high^ cells, the CD138^low^ cells showed increased uptake of 2-NBDG by 1.4, 2.5, 1.7, and 1.8-fold in MM.1S, OPM-2, U266, and H929 cells, respectively (Fig 4C). As increased glycolysis is a cellular adaptation to hypoxia [74–76] and is also relevant to clinical MM, we next cultured HMCLs in hypoxia for 3 days. A hypoxia-induced further increase in 2-NBDG uptake of 1.1, 1.9 and 1.2-fold was noted in CD138^low^ cells in OPM-2, U266, and H929 cells, respectively (Fig 4D). For the CD138^high^ cells, a comparable 2-NBDG fluorescence was noted for hypoxia and normoxia cultures in OPM-2, H929, and U266 cell lines while glucose uptake decreased for MM.1S cells cultured under hypoxia indicating metabolism heterogeneity in different HMCLs (Fig 4D).

We next determined if 2-DG treatment increases steady-state levels of pro-oxidant in MM cells. For this, we measured oxidation of DHE probe as a surrogate for O_2_^•−^ levels [31]. Consistent with the H_2_DCF-DA (Fig 3A) and PO-1 results (Fig 3B), CD138^low^ cells showed lower endogenous DHE oxidation compared to CD138^high^ cells for MM.1S and OPM-2 cells (Fig 4E), 2-DG treatment increased DHE probe oxidation in both CD138^high^ and CD138^low^ sub-populations (Fig 4E).

To understand the mechanism of 2-DG mediated oxidative stress in MM cells, we performed qPCR analysis of ER stress responsive genes, BiP (binding immunoglobulin protein/ GRP78) and ATF4 (activating transcription factor 4). Since, 2-DG is chemically 2-deoxymannose and is able to inhibit N-linked protein glycosylation [36], mannose plus 2-DG control was included. 2-DG treatment statistically increased *BiP* and *ATF4* expression in MM.1S and OPM-2 cells (Fig 4F). Notably, mannose treatment significantly reduced 2-DG mediated increase in mRNA levels of BiP and ATF4 in both MM cells (Fig 4F). Next, we examined protein expression of BiP and ER stress markers CHOP (C/EBP homologous protein). Western blotting results were agreement with the qPCR analysis where 2-DG treatment increased BiP and CHOP expression and mannose treatment significantly attenuated ER stress in both MM.1S and OPM-2 cells (Fig 4G). Overall, Fig 4 shows that CD138^low^ cells may exhibit a preferential reliance on glycolytic metabolism, and 2-DG treatment increases ER stress as well as steady-state O_2_^•−^ levels in MM cells.

### CD138^low^ cells show increased orthodox mitochondria with increased pro-oxidant levels by 10-TPP treatment

**Fig 5:**
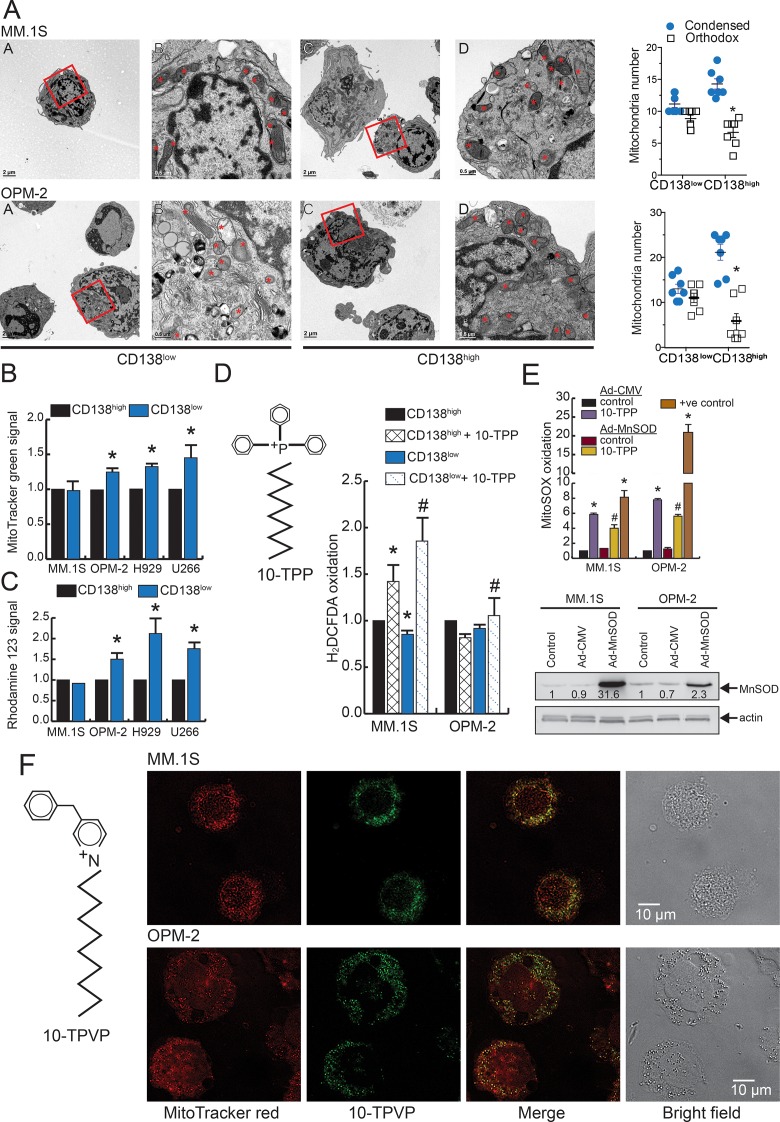
CD138^low^ cells have altered mitochondrial properties that can be utilized to induce oxidative stress by 10-TPP treatment. A. Representative electron micrographs of sorted CD138^low^ and CD138^high^ MM.1S and OPM-2 cells. For ultrastructure analysis, all mitochondria were selected (indicated by *), manually scored, and assigned either condensed or orthodox morphology. Low magnification shows the entire cell with inset used for analysis of mitochondria under higher magnification. *p < 0.01 vs. CD138^low^ cells. HMCLs were co-stained with APC-CD138 antibody and B. MitoTracker Green or C. Rhodamine 123 and analyzed by flow cytometry. Data is presented as the fold change relative to CD138^high^ cells. *p < 0.01 vs. CD138^high^ cells. D. Structure of 10-TPP; 10-TPP-induced H_2_DCF-DA oxidation in CD138^high^ and CD138^low^ cells in MM1.S and OPM-2 cells. Data of three independent runs is presented as the fold change relative to CD138^high^ cells. *p < 0.01 vs. CD138^high^ cells, ^#^p < 0.01 vs. CD138^low^ cells. E. HMCLs were transduced with Ad-CMV or Ad-MnSOD, treated with 10-TPP, and MitoSOX oxidation was analyzed by flow cytometry. Data is presented as fold change normalized to control cells expressing Ad-CMV. Antimycin A treatment was used as a positive control. *p < 0.01 vs. control cells (Ad-CMV or Ad-MnSOD), ^#^p < 0.05 vs. 10-TPP treated Ad-CMV cells. Representative Western blot of HMCLs transduced with Ad-CMV or Ad-MnSOD (MOI = 50), whole-cell extract was made at 48 h and probed with an antibody against MnSOD (24 kDa) or β-actin. The quantification of MnSOD after normalization to untreated control is shown below each band. F. Structure of 10-TPVP; representative confocal images of MM.1S and OPM-2 cells stained with MitoTracker red and 10-TPVP. The bright field view and merge image of MitoTracker Red and 10-TPVP are also shown. For panels B-E, bars represent mean of three independent runs ± SEM.

To get an idea about the overall bioenergetics of MM stem-like cells, mitochondria ultrastructure was examined using electron microscopy (CMRF, UI). For both MM.1S and OPM-2 cell lines, sorted CD138^low^ cells showed comparable numbers of orthodox (uncondensed or energized) and condensed mitochondria (Fig 5A). However, a significantly higher number of condensed (depolarized) mitochondria were noted for CD138^high^ MM.1S and OPM-2 cells (Fig 5A). Therefore, the ratio of orthodox:condensed mitochondria was increased for CD138^low^ cells (0.82 ± 0.09 and 0.92 ± 0.16 for MM.1S and OPM-2 cells, respectively) relative to CD138^high^ (0.50 ± 0.08 and 0.35 ± 0.14 for MM.1S and OPM-2 cells, respectively). Using flow cytometry, we also noted that the CD138^low^ cells had higher mitochondrial mass in OPM-2, H929 and U266 cells while the MitoTracker staining was comparable between CD138^low^ and CD138^high^ MM.1S cells (Fig 5B). R123 staining showed that relative to CD138^high^ cells, the CD138^low^ cells of OPM-2, H929, and U266 exhibit increased Δψ_m_ (Fig 5C) as also reported for other cancer stem cell types [40–42]. Overall, we noted that CD138^low^ cells have more abundant “energized/orthodox” mitochondria with higher mitochondrial mass while CD138^high^ cells show predominantly condensed “depolarized” mitochondria.

Since we measured mitochondrial differences in the bulk and stem-like cells, we next used 10-TPP to target pro-oxidant producing pathways in MM. Treatment with 10-TPP increased pro-oxidant levels in both CD138^high^ and CD138^low^ MM.1S cells by 1.4 and 1.9-fold, respectively (Fig 5D). For the OPM-2 cells, 10-TPP treatment resulted in a significant increase in H_2_DCF-DA probe oxidation in the CD138^low^ fraction. To test if 10-TPP treatment increases mitochondrial O_2_^•-^ levels, we used MitoSOX Red probe [31]. Treatment with 10-TPP significantly increased mitoSOX oxidation in both MM.1S and OPM-2 cell that were transduced with Ad-CMV. Notably, MM cells overexpressing MnSOD showed significantly lower MitoSOX oxidation (Fig 5E, upper panel). Alteration in MnSOD protein level in Ad-MnSOD transduced cells was confirmed by immunoblotting (Fig 5E, lower panel).

We utilized 10-TPVP imaging as a surrogate indicator of mitochondria localization of 10-TPP. Staining of unsorted MM.1S and OPM-2 cells with the mitochondria imaging agent (MitoTracker Red) displayed many distinct mitochondria (red fluorescence Fig 5F). Confocal analysis revealed co-localization of the 10-TPVP (blue fluorescence) with the MitoTracker Red staining, suggesting that 10-TPP may effectively localize to mitochondria in MM cells. Treatment with FCCP (mitochondrial uncoupler that dissipates proton gradient) resulted in loss of 10-TPVP signal confirming accumulation of 10-TPVP in mitochondria was driven by Δψ_m_ (data not shown).

### Combined 2-DG+10-TPP treatmfent increases selective killing of MM cells

**Fig 6:**
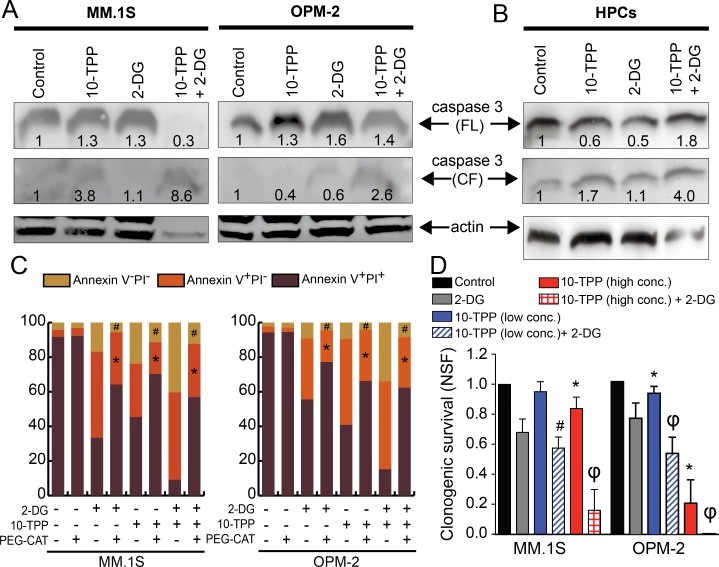
10-TPP and 2-DG treatment induces apoptosis and reduces clonogenic survival of HMCLs. A. MM1.S and OPM-2 cells or B. HPCs were treated with 10-TPP and/or 2-DG for 24 h and total protein lysate was immunoblotted for caspase 3 (full length, 37 kDa and cleaved fragment, 17 kDa). β-actin was used as a loading control. The quantification of caspase 3 after normalization to untreated control is shown below each band. C. Annexin V and PI staining of MM1.S and OPM-2 cells with 10-TPP and/or 2-DG for 12 h with or without PEG-catalase (PEG-CAT). Percentage of viable, early apoptotic (annexin V^+^ PI^-^), and late apoptotic/necrotic (annexin V^+^ PI^+^) for a representative of three independent experiments is shown. *p < 0.05 for annexin V^+^ PI^-^ fractions vs. 2-DG, TPP or 2-DG+TPP only, ^#^p < 0.05 for annexin V^+^ PI^+^ fractions vs. 2-DG, TPP or 2-DG+TPP only. D. Clonogenic assays with 2-DG and/or 10-TPP. 10-TPP low and high concentrations are 0.02/0.2 μM and 0.1/1 μM, respectively. The normalized survival fraction was calculated. Data was normalized to untreated control cells. Bars represent mean of three independent runs ± SEM. *p < 0.01 vs. control, ^#^p < 0.01 vs. 2-DG, and ϕp < 0.01 vs. 2-DG or 10-TPP.

As an indicator for activation of intrinsic apoptosis, we examined caspase 3 expression by immunoblotting (full length and cleaved fragment). In MM1.S and OPM-2 cells, densitometric analyses of immunoblots show that 10-TPP + 2-DG treatment induced significant caspase 3 cleavage compared to treatment with 10-TPP or 2-DG alone (Fig 6A). To determine therapeutic efficacy of the proposed drug combination, normal murine HPCs (sca1^-^c-Kit^-^) were used. For normal HPCs, combination of 2-DG+10-TPP showed increased cleavage of caspase 3 however, the ratio of cleaved/full length caspase 3 was comparable between 2-DG+10-TPP, 10-TPP, or 2-DG (2.2, 2.8, or 2.2, respectively, Fig 6B).

To determine the role of oxidative stress in the killing of MM cells by 2-DG and/or 10-TPP treatments, specific wells were pretreated with PEG-catalase to scavenge H_2_O_2_ [30, 77]. PEG-catalase significantly protected MM.1S and OPM-2 cells from 2-DG and/or 10-TPP-induced apoptotic death (Fig 6C). For combined 2002DDG plus 10-TPP treatment, PEG-catalase reduced early apoptotic fraction (annexin V^+^PI^-^) from ~50 to 30% and late apoptotic/necrotic fraction (annexin V^+^PI^+^) from 40 to 12% and 30 to 8% for MM.1S and OPM-2 cells, respectively (Fig 6C).

We next performed clonogenic assays to examine if 2-DG and/or 10-TPP treatment reduced clonogenic fractions in MM.1S and OPM-2 cells. Treatment with 2-DG induced ~20–30% killing of MM.1S and for OPM-2 cell line (Fig 6D). In the MM.1S cells, combination of 10-TPP (0.1 μM) with 2-DG showed significantly increased killing compared to 2-DG alone (Fig 6D). The OPM-2 cells showed ~10% and 80% killing with 10-TPP (0.2 and 1 μM, respectively) and combination with 2-DG further increased clonogenic killing compared to 10-TPP or 2-DG alone (Fig 6D). Overall, Fig 6 shows that combination of 2-DG plus 10-TPP provides a therapeutic window by showing selective cytotoxicity towards cancer and not normal cells.

## Discussion

Clinical course of MM is fraught with emergence of refractory disease and high mortality [2, 3]. A growing body of evidence suggests that a minor, therapy-resistant, clonogenic sub-population of tumor cells is most likely the principal roadblock to curing MM [4, 8]. However, the identity of these stem-like MM cells remains only partially resolved [5, 7]. Matsui *et al*. [57] identified a CD138^-ve^ memory B-cell like clonogenic fractions in HMCLs and clinical MM samples. Besides CD138 expression, functional markers such as SP staining and aldehyde dehydrogenase activity (Aldefluor assay), mRNA expression of induced pluripotent stem cells genes (i.e. OCT4, NANOG, SOX2), drug resistance and chromosomal instability genes (i.e. NEK2, BTK, RARα2), *BCRP*, and/or *ALDH1A1* expression has been used to identify stem-like MM cells [7, 8]. In the present study we have used two established markers of MM stem-like cells (CD138 immunophenotyping and SP staining) to characterize the oxidative metabolic properties of stem-like cells.

Based on *CD138*, *OCT4*, *NANOG* expression, we report that culturing cells under hypoxic conditions results in “increased MM stemness”. We have focused on hypoxia as it is the primary regulator of stemness for normal adult stem cells and CSCs [78], constitutes a specific feature of MM bone marrow [79, 80], and is being used as therapeutic target for MM [81]. Moreover, hypoxic-inducing factors (HIFs, HIF-1α and HIF-2α) are stabilized in MM patients with prognostic relevance [82]. Also, HIF-1 contributes to metabolic shift via upregulation of glucose transporters and glycolytic enzymes, and can aid in re-establishing redox homeostasis and cell survival under prolonged hypoxia [83]. However, besides hypoxia, CD138 down-regulation on MM cells within the bone marrow microenvironment can also occur via interaction with bone marrow stromal cells and interleukin 6 [61, 84, 85].

New approaches for targeting MM stem-like cells are under development like inhibitors of RARα2 or its downstream Wnt and Hedgehog signaling pathways and bruton tyrosine kinase (BTK) inhibitor [63, 86]. We have previously utilized redox-based chemotherapeutics to elevate mitochondrial ROS levels and attain selective killing of MM cells [30, 31]. We have also published on the role of antioxidant network (endogenous or driven by IL6−NFκB pathway in intrinsic or acquired drug resistance [31, 32]. In this study we investigated the unique pharmacological targets, related to the oxidative metabolism, in MM stem-like cells such that oxidative stress-mediated cytotoxicity can be selectively induced in the drug-resistant tumor cells relative to normal stem cells. Recent studies have provided insight on the redox status of CSCs where low levels of intracellular ROS are reported in the CSC fractions of leukemia, breast and prostate cancer [87–89]. We detected low steady-state pro-oxidant levels in stem like MM cells (CD138^low^ vs. CD138^high^ cells and SP vs. MP cells). CSCs can maintain lower ROS levels via a preferential reliance on glycolytic metabolism [90–95]. We found that the CD138^low^ cells have increased 2-NBDG uptake relative to the bulk MM cells. We will extend these studies by performing real-time measurement of mitochondrial respiration and glycolysis (Seahorse Flux Analyzer) in MM stem-like cells enriched form cell lines and patient samples. Besides increased glycolysis, increased expression of antioxidant systems can also confer lower ROS levels in CSCs [88, 96]. Ongoing studies in the lab are utilizing sorted stem-like cells to perform mRNA analysis of thiol regulatory proteins.

Bulk MM cells have been reported to overexpress glucose transporters and to undergo apoptosis upon pharmacological inhibition of glucose metabolism [16–20]. However, targeted inhibition of glycolytic metabolism in MM stem-like cells for therapy has remained unexplored. Microarray results with human MM biopsies suggest that increased expression of glycolytic genes may predict poorer overall survival outcomes. However, a tight correlation between gene transcription and pathway functionality may always not hold true [97]. Further studies in primary MM samples are required to show a direct correlation between glycolysis and overall survival outcomes.

Based on the observation that MM stem-like cells have lower steady-state ROS levels and increased 2-NBDG uptake, we selected 2-DG to inhibit the glycolytic pathway. We used a 2-DG concentration (20 mM) [98] that is in excess to the glucose concentration in the cell culture media (RPMI-1640, 11 mM glucose). In HMCLs, 2-DG treatment increased steady-state levels of O_2_^•−^ in both stem-like and bulk tumor cells. Pharmacological manipulation of glucose metabolism (using dichloroacetate that inhibits pyruvate dehydrogenase kinase) has been shown to incur specific cytotoxicity on glioma stem cells and not normal neural stem cells [92]. Biochemically, 2-DG blocks pyruvate production by glycolysis and partially inhibits the pentose phosphate pathway, reducing NADPH regeneration by 50% and increasing cellular oxidative stress in cancer cells [99, 100]. Also, 2-DG treatment induces ER stress via inhibiting N-linked protein glycosylation [36]. Notably, MM is a plasma cell neoplasm with extensive ER network required for antibody secretion [101] and undergoes unfolded protein response activation and cell death by prolonged ER stress [102, 103]. Also, high ER function in MM has been shown to result in an increased passive leak of Ca^2+^ into cytoplasm [104]. Constitutive Ca^2+^ transfer from the ER to mitochondria is essential for maintenance of cellular bioenergetics [105]. Therefore, besides inhibiting oxidative metabolism, 2-DG treatment in MM may also disrupt ER−mitochondrial Ca^2+^ homeostasis. This may result in a feed-forward cycle [106, 107], enhancing oxidative stress-mediated killing of stem cells in combination with other mitochondrial redox-modulating chemotherapies.

In this study, we have used unsorted cells, stained for CD138 expression to differentiate between mature and stem-like MM cells, and directly compare glucose uptake, mitochondrial density, and mitochondrial membrane potential in these sub-populations. To better characterize differences in glycolytic rate and OXPHOS capacity, further studies are required to assess differences in glycolytic rates (lactate plus pyruvate formation) and OXPHOS capacity (oligomycin sensitive oxygen consumption) in stem-like vs. bulk MM cells. Also, it remains to be seen if CD138^low^ cells show dysregulated expression of PGC-1α (peroxisome proliferator-activated receptor γ coactivator 1) resulting in increased mitochondrial mass. Notably, altered PGC-1α expression has been linked to metabolic adaptation and survival of cancer stem cells [108, 109].

Recent reports showing that normal stem cells and induced pluripotent stem cells retain young mitochondria with high metabolic capacity [54, 110, 111]. Similarly, physiological aging may occur in cancer where CSCs show hyperpolarized Δψ_m_ [24, 44, 45]. We report that the CD138^low^ stem-like cells have increased mitochondria numbers, Δψ_m_, and also higher percentages of mitochondria with orthodox configuration. It can be speculated that these mitochondrial properties of stem-like cells could be linked to increased metabolic demands for differentiation to mature MM cells that are more proliferative and highly secretory cells. Further studies using primary clinical samples may be useful to validate the changes in mitochondrial properties seen in stem-like cells of HMCLs.

Several mitochondria-targeted agents have developed by using the synthetic lipophilic TPP cation that can selectivity target cancer cells relative to normal cells [39]. Screening of a commercially available library of 46 different TPP-based compounds (specs.net) provided 10-TPP as one of the lead agents with high and selective cytotoxicity in melanoma cell lines (unpublished results). 10-TPP (C_28_H_36_BrP, 483.5) contains an aliphatic 10-carbon chain that is believed to uncouple mitochondria via embedding in the inner mitochondrial membrane [38]. A TPP derivative Mito-VES (vitamin E succinate) has shown selectivity towards breast cancer cells via mitochondrial localization and increasing ROS levels from ETC complex II [112]. Also, Mito-VES with a 11 carbon aliphatic chain length is most effective in mitochondrial ROS generation (compared to 5, 7 or 9 carbon linker) [43]. Also molecular modeling of Mito-VES indicated that TPP^+^ anchors at the matrix face of the inner mitochondrial membrane while 11-C linker enables a close positioning of VES with the UbQ site of complex II [112]. Based on mitochondrial alterations in MM stem like cells (increase mitochondrial number, Δψ_m_, and orthodox:condensed mitochondria ratios) we hypothesized that 10-TPP treatment can potentially increase oxidative stress and cytotoxicity of MM stem-like cells. For mitochondrial imaging, we used an aggregation-induced emission active triphenylvinylpyridine (TPVP) agent with 10-carbon chain (10-TPVP, C_35_H_40_BrN, 554.6) [53, 113]. Our results suggest that 10-TPP can potentially localize to mitochondria and increases cellular pro-oxidant levels as well as mitochondrial O_2_^•−^ level in MM cells.

It is established that tumor cells often maintain redox homeostasis via multiple pathways and can adapt to chronic metabolic oxidative stress [114]. We therefore used a two-pronged approach that simultaneously targets oxidative (2-DG and 10-TPP) and glycolytic metabolism (2-DG) to reduce clonogenic fraction in MM cell lines presumably via metabolic oxidative stress-mediated cytotoxicity. To determine therapeutic efficacy of the proposed drug combinations, we used normal HPCs and noted that the DG+10-TPP treatment was minimally toxic towards these normal cells. Clinically, 2-DG is well-tolerated in humans and is being actively pursued as an adjuvant to cancer chemotherapy [33, 115]. However, chronic ingestion of 2-DG in rats showed increased mortality with cardiac pathology [116]. Therefore, clinical development of 2-DG for MM therapy will require careful assessment of potential long-term normal tissue toxicity. Also, 10-TPP has a similar chemical structure as mitochondrial CoQ10 (MitoQ) that has been considered safe for long-term administration in human trials [117, 118]. In preliminary studies, oral dosing of 10-TPP (100 μM for 17 days) caused no evidence of hematological toxicity in mice (data not shown). By *in vivo* limiting dilution assay, future studies will combine 2-DG (400 mg/kg intraperiponeally 5 days per week for 2 weeks)[119] with 10-TPP to determine if combination therapy can deplete MM clonogenic fractions and improve therapy outcome.

In conclusion, we show that altered oxidative metabolism can be used as a novel indicator of stem-like cells in MM. We report that MM stem-like cells may have a preferential reliance on glycolytic metabolism with increased mitochondrial numbers, Δψ_m_, and orthodox mitochondrial configuration. The proposed biochemical approach of combining 2-DG with 10-TPP is expected to provide a therapeutic window to selectively eliminate MM and drug-resistant residual disease while sparing normal cells, leading to more effective MM therapy.
